# National spine surgery registries’ characteristics and aims: globally accepted standards have yet to be met. Results of a scoping review and a complementary survey

**DOI:** 10.1186/s10195-023-00732-4

**Published:** 2023-09-16

**Authors:** Simona Pascucci, Francesco Langella, Michela Franzò, Marco Giovanni Tesse, Enrico Ciminello, Alessia Biondi, Eugenio Carrani, Letizia Sampaolo, Gustavo Zanoli, Pedro Berjano, Marina Torre

**Affiliations:** 1 Scientific Secretariat of the President’s Office, Italian National Institute of Health, Istituto Superiore di Sanità, Viale Regina Elena, 299, 00161 Rome, Italy; 2 Department of Mechanical and Aerospace Engineering, La Sapienza University of Rome, Rome, Italy; 3 IRCCS Ospedale Galeazzi-Sant’Ambrogio, Milan, Italy; 4 Department of Medico-Surgical Sciences and Biotechnologies, Rome, Italy; 5 Orthopaedics Section, Department of Neuroscience and Organs of Sense, Faculty of Medicine and Surgery, University of Bari, AOU Consorziale Policlinico, 70124 Bari, Italy; 6 National Centre for Disease Prevention and Health Promotion, Italian National Institute of Health, Rome, Italy; 7 Casa Di Cura Santa Maria Maddalena, Occhiobello, Italy

**Keywords:** Registry, Spine, Medical device, Review, Musculoskeletal disease, Back pain

## Abstract

**Background:**

Surgery involving implantable devices is widely used to solve several health issues. National registries are essential tools for implantable device surveillance and vigilance. In 2017, the European Union encouraged Member States to establish “registries and databanks for specific types of devices” to evaluate device safety and performance and ensure their traceability. Spine-implantable devices significantly impact patient safety and public health; spine registries might help improve surgical outcomes. This study aimed to map existing national spine surgery registries and highlight their features and organisational standards to provide an essential reference for establishing other national registries.

**Methods:**

A scoping search was performed using the Embase, PubMed/Medline, Scopus, and Web of Science databases for the terms “registry”, “register”, “implantable”, and all terms and synonyms related to spinal diseases and national registries in publications from January 2000 to December 2020. This search was later updated and finalised through a web search and an ad hoc survey to collect further detailed information.

**Results:**

Sixty-two peer-reviewed articles were included, which were related to seven national spine registries, six of which were currently active. Three additional active national registries were found through the web search. The nine selected national registries were set up between 1998 and 2021. They collect data on the procedure and use patient-reported outcome measures (PROMs) for the follow-up.

**Conclusion:**

Our study identified nine currently active national spine surgery registries. However, globally accepted standards for developing a national registry of spine surgery are yet to be established. Therefore, an international effort to increase result comparability across registries is highly advisable. We hope the recent initiative from the Orthopaedic Data Evaluation Panel (ODEP) to establish an international collaboration will meet these needs.

**Supplementary Information:**

The online version contains supplementary material available at 10.1186/s10195-023-00732-4.

## Introduction

Surgery involving implantable devices is widely used to solve several health issues. Cardiac implantable devices, joint prostheses and spine implants are recognised to impact patients' health positively. They help to restore the body's functionality, relieve pain, and improve quality of life [1–3]. On the other hand, such devices may lead to unforeseen and unwanted side effects related to the implanted device thus implying a risk to the patient’s safety [4, 5].

A *patient registry* is defined as “an organised system that uses observational study methods to collect uniform data (clinical and other) to evaluate specified outcomes for a population defined by a particular disease, condition, or exposure, and that serves one or more predetermined scientific, clinical, or policy purposes” [6]. Moreover, a *registry* is also defined as an “organised system with a primary aim to increase the knowledge on medical devices contributing to improve the quality of patient care that continuously collects relevant data, evaluates meaningful outcomes and comprehensively covers the population defined by exposure to particular device(s) at a reasonably generalisable scale (e.g., international, national, regional, and health system)” [7]. Registries can be local or maintained by a single institution with multiple locations; in this case, they usually collect enormous amounts of data to inform the local clinical government. In contrast, regional or national registries collect limited high-quality data encompassing the whole picture. The two approaches are complementary. Local registries have delivered helpful information for quality improvement by several organisations [8]; nevertheless, speaking from a scientific perspective, only national registries covering a vast, unselected population can provide reliable scientific evidence that can be transferred to different contexts and countries [9].

This study aimed to map existing national spine surgery registries and highlight their features in terms of data collection and organisational standards as an essential reference for the future establishment of the Italian Spine Registry (Registro Italiano Dispositivi Impiantabili per chirurgia Spinale, RIDIS) and other national registries.

## Methods

### Study design

The study was designed as a scoping literature review followed by a web search to map and analyse existing active national spine registries and determine their organisational structures and scientific production. The scoping review’s design, searches and reporting stages were realised according to the Preferred Reporting Items for Systematic Reviews and Meta-Analyses (PRISMA) guidelines [10]. An ad hoc survey to collect additional information was also designed and sent to the directors of the registries selected from the literature review.

### Literature search

The key review question was: “what, and how many, national spine surgery registries exist and are currently active worldwide?”. The aim was to retrieve online biomedical literature published by spine registries worldwide and select those active nationally. The expert panel set the inclusion/exclusion criteria to get articles with any study design published from January 2000 to December 2020 (observed period of the literature search) in any foreign language besides Italian. The online search covered the highly structured peer-reviewed databases PubMed/Medline, Embase, Scopus, and Web of Science, and was inspired by the systematic review performed by Van Hooff in 2015 [11]. The core search included the terms “registry”, “register”, “implantable”, and all the potential synonyms and terms related to spine diseases and national registries. The search string is given in Additional File 1.

The studies identified through the search were uploaded to Mendeley (Mendeley Ltd, Elsevier, London 2019), which was used for managing references and removing duplicates. Two authors (MF and SP) independently reviewed each article’s title and abstract and selected the eligible studies according to the inclusion and exclusion criteria set. A third author (FL) resolved discrepancies. Furthermore, the reference list of the selected studies was screened to find any relevant additional original articles. Based on the experts’ suggestions, an independent web hand search was also performed to find articles of interest that were not detected by the online search.

### Inclusion and exclusion criteria

Studies were included if they (1) considered spine surgery, (2) were observational studies (cohort studies, case–control studies, case series) or randomised or quasi-randomised clinical studies with human subjects and without restrictions regarding the date of publication, and (3) considered named active national registries.

Studies were excluded if they (1) did not relate to spine surgery, (2) presented data that did not come from an active national spine registry, (3) were limited to single regions, scientific societies, or single health organisations, (4) were audit studies or conference reports, (5) did not involve humans, (6) were in vitro studies, (7) were articles published in abstract form only or for which the title/abstract was unavailable, and (8) were patient registries.

### Supplementary web search and survey to verify the registries’ status

A supplementary web search (for a landing page or website) was performed to verify the active status of the selected national registries and get specific information on their clinical and management characteristics and facilities. Their official websites and articles or reports were checked. Missing information was later obtained through an ad hoc survey emailed to the authors of the selected publications or the institutions hosting the registry. The information collected was then summarised in a standardised form.

## Results

**Fig. 1:**
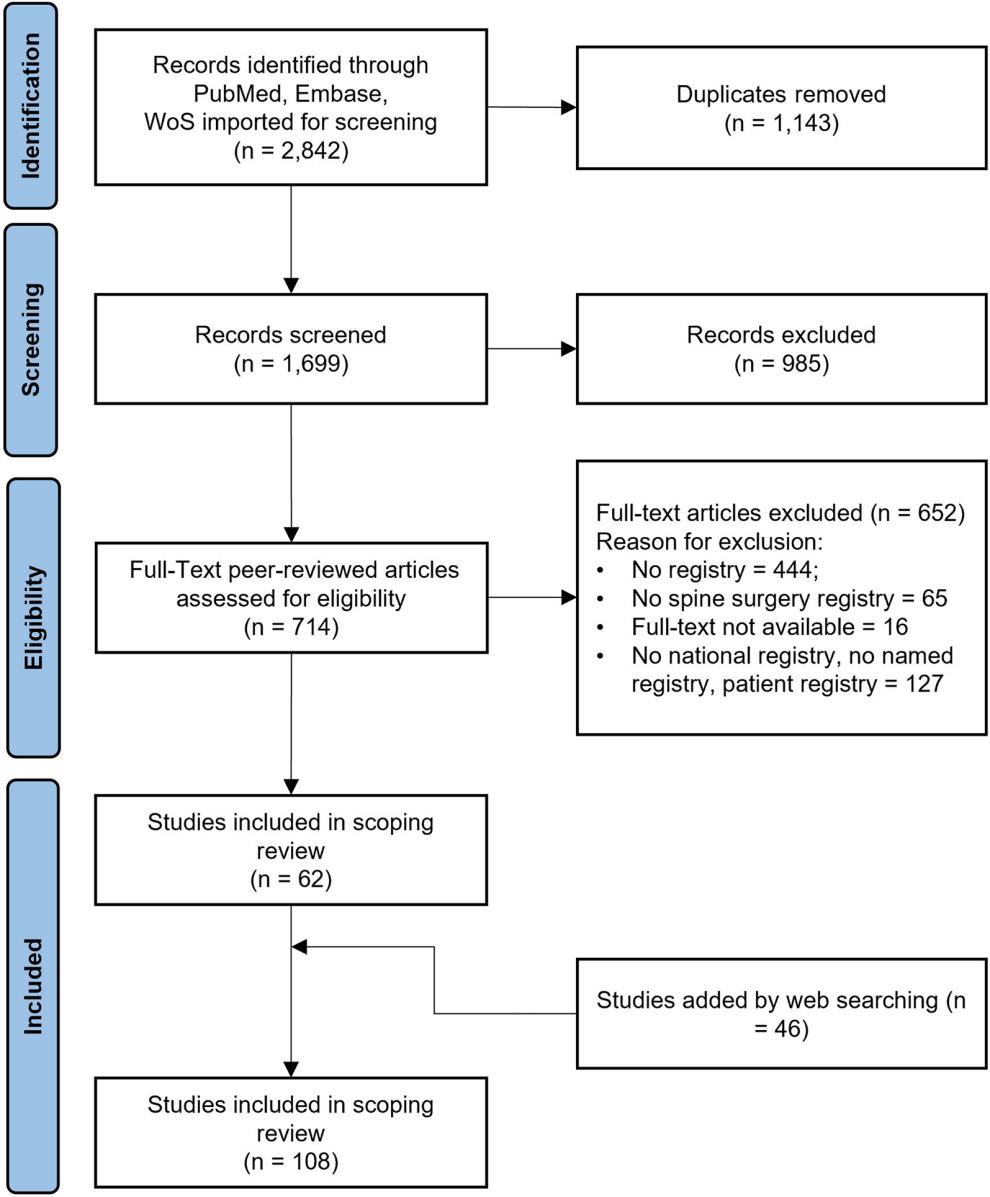
Studies inclusion flow chart (PRISMA: Preferred Reporting Items for Systematic Reviews and Meta-Analyses)

After duplicate removal, the search yielded 1,699 articles. Of those, 985 were excluded based on title and abstract and 652 more after full-text reading. The residual 62 peer-reviewed articles were selected for inclusion. After a website search and an independent hand search, 46 studies were included in the scoping review. The study search and selection are presented as a PRISMA flow chart in Fig. 1.

The authors MF and SP agreed on about 75% of the selection. A consensus was reached for the residual 25% with  the involvement of the third investigator (FL).

The scoping review identified seven nation-based spine registries: the British Spine Registry (BSR), the Canadian Spine Outcomes and Research Network (CSORN), the Danish Spine Registry (DaneSpine), the German Spine Registry (DWG), the Norwegian Registry for Spine Surgery (NORspine), the Swedish Spine Registry (Swespine), and the Swiss Spine Registry (SWISSspine).

Table 1 shows the number of papers included in the review from the database search and those selected from the web search by the registry. The list of the 108 articles included in the review is presented in Lists S1 and S2 of Additional File 1.

**Table 1:** Number of publications included from the online database and web hand searches by registry name

Registry involved in the study	No. of papers included from the online database search	No. of papers included from the web hand search	Total
British Spine Registry (BSR)	3	1	4
Canadian Spine Outcome and Research Network (CSORN)	2	10	12
Danish Spine Registry (DaneSpine)	4	5	9
German Spine Registry (DWG)	1	6	7
Norwegian Registry for Spine Surgery (NORspine)	6	7	13
Swedish Spine Registry (Swespine)	33	14	47
Swiss Spine Registry (SWISSspine)	11	3	14
*Total no. of included papers* *referring to* *only one* *registry*	*60*	*46*	*106*
*Total number of included papers* *referring to* *more than one registry*	*2*	*-*	*2*
*Total number of papers included*	*62*	*46*	*108*

**Fig. 2:**
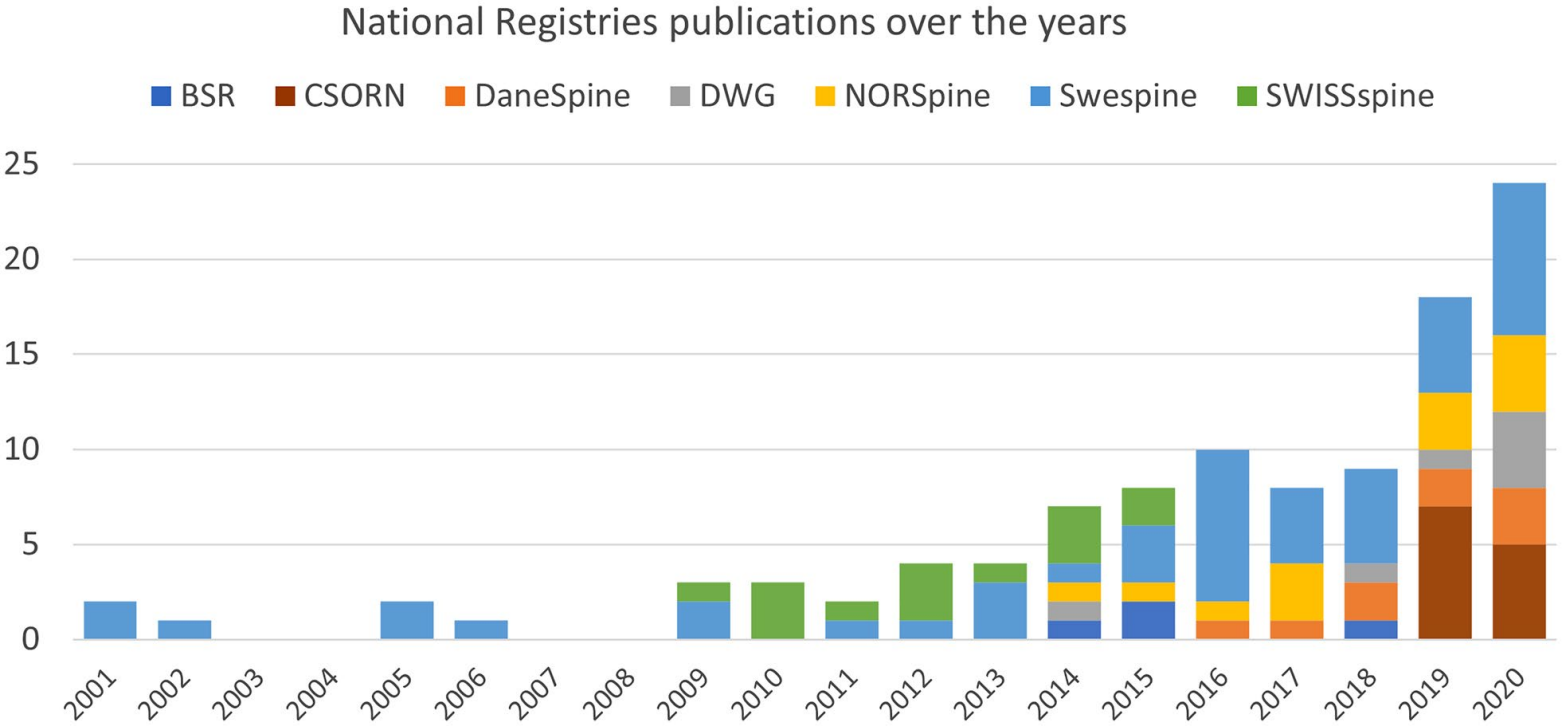
Distribution of the selected papers related to only one registry, by registry and year of the period observed by the literature search

Figure 2 shows the selected papers distribution by registry and year of the observed period considered by the literature search. Swespine  was the first to appear on the international scene in 1998. Since then, there has been widespread rising interest in spine registries. A growing number of scientific publications then followed the increase in the number of national spine registries.

The supplementary web search and cross-checking of the information identified the following five additional national registries: the Australian Spine Registry; the Dutch Spine Registry (DSSR); the Finnish Registry (FinSpine); the RIDIS and the Swiss National Implant Registry, Spine (SIRIS Spine). The latter, which is currently active and uses the structure designed for Spine Tango, was started in 2021 to replace SWISSspine  (identified by the literature search), which shut down around 2014. The DSSR was discontinued on 21 December 2020 [12] and is now in its starting phase (Carmen L. A. Vleggeert-Lankamp, personal communication to Gustavo Zanoli, email dated 05/09/2022). It was not included in the first analysis, like the Italian Spine Registry (RIDIS) [13], because it is not yet active.

Our research finally identified 12 spine registries based on a national setting, with nine currently active. Specifically, seven were identified by the comprehensive literature search and five by the supplementary web search done to update and complete the first online search performed.

### Synthesis of findings

The nine active registries’ structural and operational characteristics are presented in Table 2, while the clinical data collected are reported in Table 3. A list of the acronyms mentioned in the paper is reported in Abbreviations section  1.

**Table 2:** Structural and operational characteristics of the registries included in the review

Registry name	Australian Spine Registry	BSR	CSORN	DaneSpine	DWG	FinSpine	NORspine	Swespine	SIRIS Spine
Country	Australia	United Kingdom	Canada	Denmark	Germany and Austria	Finland	Norway	Sweden	Switzerland
Year established	2018	2012	2015	2009	2011*	2016	2006	1998	2021
Source of funding	No stable funding; intermittent short-term funding agreements with a variety of supporters; Australian government provided partial 2-year funding for 2022/2023*	The British Association of Spine Surgeons (BASS)	CSREF; CSS; industry and private donations	Public*	German Spine Society	Public*	Helse Nord RHF; UNN HF	Public No stable funding; must apply to “Sveriges Kommuner och Regioner” (SKR) for funding each year*	Initial funding provided by the SIRIS Foundation. Today, hospitals pay a specific fee for every single case, part of which is used to run the registry*
Coverage (%)^a^	NA	NA	50%	80%*	NA	83% public hospitals*	100% lumbar 91% cervical*	98%*	100%
Completeness (%)^b^	> 80%*	80%*	10%	NA	NA	80%*	81% lumbar 75% cervical*	86%*	NA
Data quality monitoring process or certification	Partial audit of 15% of private practice data before Covid pandemic*	Amplitude/steering committee*	The National Data Quality Coordinator	NA	Yes	Comparison to previous data uploads; validation of the data by clinical experts and descriptive statistics*	Yes	Steering group	Still in development; will be based on the surgery coded for financial reimbursement of the hospitals*
Website available? (language)	Yes (English)	Yes (English)	Yes (English)	Yes (Danish)	Yes (German)	No	Yes (Norwegian)	Yes (Swedish)	Yes (English, German, French and Italian)
Reports (language)	Yes (English)	Yes (English)	Yes (English)	Yes (Danish)	Yes (German)	No*	Yes (Norwegian)	Yes (Swedish, English)	No*
Are reports freely available?	Yes	Yes	Yes	Yes	Yes	No	Yes	Yes	Yes, although the first report is expected in 2024*
Frequency	Annual	Annual	Annual	Annual	Annual	Annual*	Annual	Annual	Annual*
Methods of data collection (text)	Primarily email; questionnaires through the KEOPs database web-based portal; paper-based by mail where necessary	Paper and electronic Paper forms collected locally and uploaded electronically	Paper forms and direct online entry; paper forms collected locally and uploaded electronically	Questionnaire entered into the database of the reporting clinics; patient-reported data entered directly into the database. Stored by Public Health in one database for the whole country*	Direct online entry; Operation 2017 form	Direct online entry*	Self-administered and identical questionnaire or direct online entry	Web; paper*	Online only*
Administered questionnaires (PROMs, clinical, PREMs)	EQ-5D-3L; EQ-VAS	EQ-5D; Fear-Avoidance; DASS-21; FABQ; PHQ2; PHQ9; ZSDS; PSFS; mJOA, JOA, PGIC, PSFS; SBI; SFI; MSPQ*	EQ-5D;; PHQ9; ODI; NDI mJOA, LANSS, SRS22*	EQ-5D; SF-36; SRS22; HRQOL score	SF-36; EuroQoL-5D	EQ-5D-3L; PREM*	EQ-5D; EQ-VAS	EQ-5D; EQ-VAS; SRS-22r	PROMs are piloted and will presumably go live in Jan 2024
Timing of post-operative assessment	3 and 6 mo; 1 and 2 y	6 weeks; 6 mo; 1 and 2 y	[Baseline peri-operative]; 3 mo; 1,2 and 5 y*	[Baseline + pre-operative]; 1,2,5 and 10 y	[Baseline + peri-operative]; 3 mo; 1 and 2 y	3 mo; 1,2,5 and 10 y*	[Baseline + peri-operative]; 3 mo; 1 y and then longer term where necessary*	1,2,5 and 10 y	To be implemented starting from 2024: 3 mo; 1 y*

* Data obtained or confirmed by the survey

^a^ Number of hospitals participants/total number of hospitals performing spine surgery in the country

^b^ Number of surgeries registered/number of surgeries performed in all the hospitals in the country

**Table 3:** Spine conditions and clinical data collected (pre-, post- and peri-operative)

Registry name	Australian Spine Registry	BSR	CSORN	DaneSpine	DWG	FinSpine	NORspine	Swespine	SIRIS Spine
Statistical unit of analysis	Procedures/patients	Patients	Patients	Patients	Patients	NA	Procedures/patients	Procedures/patients	NA
Spine condition evaluated	D; S	LD; ST + DE; STR; SI*	D; DH; DE; DDD; DS; SL; SP; SS; ST; STR	DI; DDD; DH; SDR; SD; SF; SL; SP; SPL; SS; ST	All conditions of the spine*	Whole spine and all conditions*	CD; LD*	BP; DH; DDD; SL; SS; CD; LD; DE; SI; ST*	Degenerative diseases; non-degenerative SPO; RE; K; V*
Functional status	ODI; NDI	ODI; JOA*	ODI; NDI	ODI; NDI; self-rated walking distance	NDI; COMI; ODI	ODI; NDI*	ODI 2.0; NDI	ODI; NDI; self-rated walking distance	Any
Pain	Not collected*	VAS back; VAS leg; NDI; MDI; SSS; NRS; Pain Catastrophizing Scale; BPI; COMI; PSAS*	NRS leg; NRS back; NRS neck; NRS arm	VAS (arm, neck, back, leg)	VAS leg, back; RO	VAS neck, arm, back, leg; pre-operative duration of pain*	NRS back/leg; NRS neck/arm + headache; European myelopathy score (EMS, c-spine)*	NRS leg/back; GA back/leg; pre-operative duration of pain (0-3 m/3–12/12–24/ > 24); arm and neck pain	Any
Pre-operative data (demographic and patient condition)	Age; sex; comorbidity; thoraco-lumbar, cervical, and deformity	Demographics; clinical features; neurological findings; comorbidities; imaging; pathology*	Age; sex; BMI; previous surgery; marital status; smoking; education; exercise; work status; compensation; healthcare resources; imaging; medication; expectations; comorbidities	Patient-reported outcome measures, demographic*	Age; sex; BMI; smoking; risk factors; comorbidity; pre-treatment; medical factors; psycho-psychosocial or behavioural factors; lawsuits; drug abuse; socio-economic; work status	Comorbidities; work status; pain; medication; height; weight; smoking*	Patient-reported outcome measures; demographics; lifestyle	Sex; smoking; previous surgery; work status; comorbidity; walking distance; physiotherapy; antimorphic*	Age; sex; smoking; number of previous surgeries (any level); height; weight; BMI*
Peri-operative data (surgical and before discharge)	Level; KEOPS screen grab	Surgical details; implants*	ASA; surgery type; implants; surgery duration; blood loss; operative level*	Diagnosis; type of surgery; implant; side; level; antibiotic; hospital stay; bone graft; revision surgery*	Lesion; previous surgery; level; reoperation; access; technology; operation time; blood loss; transfusion; stabilisation	Diagnosis; type of surgery; pre-operative neurological deficits; complications; level of operation; antibiotic; thrombose prophylaxis; hospital stay; bone graft; cement augmentation; revision surgery*	Medication; previous surgery; imaging; diagnosis; treatment; comorbidity; work status; symptoms; operation time; hospital stay; surgery type*	Diagnosis; type of surgery; implant; side; level; antibiotic; hospital stay; bone graft; imaging; revision surgery*	Diagnosis; level; type of surgery; ASA status; anaesthesia (K, V); access and surgery instructed and/or accompanied by a senior consultant (degenerative diseases or non-degenerative SPO or RE); materials (fusions) *
Complications	Collected by surgeon screen grab*(list not provided with the survey)	Implant malposition—removed/ replaced; spinal cord injury; vascular injury; CVA; blindness; wrong level; wrong side; dural tear; cardiac event; death; nerve injury; radicular pain/ weakness; lymphatic injury; dysphonia; excessive bleeding; cauda equina injury; peripheral neuropraxia; embolism; dysphagia*	Airway; allergic reaction; dural tear; cutaneous injury; hypotension; implant malposition—removed/ replaced; excessive bleeding; neural injury; vascular injury; cardiac event; cerebrovascular event; delirium; dysphagia; fall; GI bleeding; haematoma; bowel obstruction; infection; neurological deterioration; pneumonia; renal insufficiency; embolism; urinary retention; wound dehiscence/drainage*	Embolism; vein thrombosis; haematoma; Homer's syndrome; dural tear; urinary infection; cauda equina injury; spinal cord injury; vocal cord injury; wound infection; urinary retention*	Peri-operative, early post-op, and post-operative	Peri-operative, immediate post-operative, late post-operative*	Peri-operative; post-operative; 3-month follow-up; re-operations at 90 days; 30-day mortality*	Peri-operative; post-operative; 1 y follow-up	Information collected by the registry* (list not provided with the survey)
Others			Independence, physical capacity, recreation; social contacts; mental well being; balance, numbness, satisfaction		Hospital stay; operation time; ASA		Hospital stay; operation time; GPE; satisfaction; ASA*	Satisfaction (Likert scale); gait speed (10MWT); quadriceps strength; analgesics	

* Data obtained or confirmed by the survey

### Structural and operational characteristics

The nine national registries included in the review were set up between 1998 and 2021. Some are managed and funded by clinician associations, while all the Scandinavian registries (DaneSpine, FinSpine, NORspine, Swespine) are publicly funded. The Australian and Canadian registries receive different types of funding, including industrial funds. Only some of the registries included have coverage and/or completeness data to provide. For all the registries selected in this review, the annual report is the most widely used reporting resource and is generally publicly available. Moreover, they base their data collection on hybrid systems that collect data from paper records and emails or through a secure web-based system, recording PROMs in electronic format and querying administrative databases (Table 2).

### Clinical data collected

All registries consider the main patient-reported outcome domains and mainly use the Oswestry Disability Index (ODI), Numeric Rating Scale (NRS) and EuroQol-5D (EQ5D). Most of them also record clinical outcomes such as complications and reoperations. All have at least 24-month follow-up assessments, except for NORspine and SIRIS Spine (12 months). Some registries consider longer follow-up assessments (5 years CSORN; 10 year DaneSpine, Swespine, FinSpine) (Table 2). 

## Discussion

In surgical fields with high rates of technological innovation and in which new devices are constantly available, it is often challenging to obtain high-quality evidence, not only because of the intrinsic difficulties of running a surgical randomised controlled trial (RCT), but also because the timeframe and sample size required to assess clinically significant outcomes (especially harms) may hinder the conduct of meaningful clinical trials. RCTs might provide a higher quality level of evidence than registries do but they have limitations, such as very high costs, the time required, and the difficulty involved in conducting the study [14]. From a technical point of view, observational studies represent the vast majority of registry-based studies. If appropriately validated, registry data can provide statistical measures of real-world health status with valuable societal benefits [15]. They can also monitor surgical activities by keeping track of  outcomes and of continuous updates in techniques used for surgical procedures  [16]. Like any clinical study, registry studies must follow strict methodological rules to be valid and informative, since only a well-structured data collection allows solid population-based epidemiological and statistical analyses [17]. Implant registries for orthopaedic surgery aim to collect information on patients, implants, and procedures and to assess surgical methods as well as types of implants and their materials, design, and other features. Coverage and completeness are the main parameters used to evaluate the quality of registries and their ability to provide reliable feedback [18]. High levels of coverage and completeness imply the ability to detect the performance of a wide range of devices and surgical techniques used in the whole population in real-life situations, thus partly overcoming the selection bias that could play a significant role in the absence of randomisation. While differences in patient selection, surgeon ability, and organisational contexts can hinder the possibility of a fair comparison of devices in terms of effectiveness (i.e. determining which is the best implant) when considering a limited context, the large scale of registries allows quick detection of harms (i.e. the rapid identification of underperforming implants) [19].

A successful registry can promptly detect device failures [20] during the follow-up. Failure is considered the gold standard in joint replacement registries when both patient and surgeon have agreed that reoperation is needed because of a dramatic decrease in the patient’s quality of life. Spine surgery is a relatively new and ultra-specialised branch, with considerable evidence obtained on it in recent years. In spine surgery, however, reoperation does not necessarily indicate implant failure, as implant removal is sometimes the standard clinical procedure (e.g. in the case of fractures). Moreover, the challenge faced by spine surgery registries is further complicated by the anatomical and functional complexity of the spine, the nature of the different conditions treated with spine surgery, the high number of available implants, and the need to both collect more variables and consider other outcomes when assessing the patient’s quality-of-life improvement; these factors require a more global and complex approach than arthroplasty registries accomplish [21].

The inclusion criteria for our review, based on those of Malchau [9], were strictly established to select only national-based registries. This specific criterion caused the exclusion of registries that, although well founded, were multicentre and therefore did not cover a national population. Specifically, Kaiser Permanente (an American integrated managed care consortium), the National Neurosurgery Quality and Outcomes Database (N(2)QOD) (developed by the American Association of Neurological Surgeons), NeuroPoint (the NeuroPoint Alliance program to improve the quality of care), the National Surgical Quality Improvement Program (NSQIP) Registry and the American Spine Registry (a collaborative effort between the American Association of Neurological Surgeons and the American Academy of Orthopaedic Surgeons) are volunteer-based and only enrol patients from several areas of the United States. As for Spine Tango, although it is internationally recognised as a well-structured registry, it was excluded because it was conceived as a multinational state registry. Despite this, Spine Tango’s design and organisation could be worthwhile looking into, and we consider it a valuable source of inspiration for national registries, as has happened in Switzerland. In general, the scientific production of registries is hugely varied and diversely significant. It also includes reports that are usually available on the registries’ websites but are rarely indexed in peer-reviewed databases, so they can only be searched for manually. Thanks to a thorough web search, it was possible to identify three more active registries that were not otherwise detected by the scoping review.

Forty-six out of 108 papers based their main research question on pre-operative and post-operative patient questionnaires to evaluate the efficacy of surgery or carry out epidemiological studies on the population. Some of these articles have laid solid foundations upon which, even today, the effectiveness of spine surgery is based [22–24]. Many analysed the complication rate and compared the clinical efficacies of different surgical procedures. Many others compared other surgical procedures in terms of clinical effectiveness and analysed the complication rate, paving the way for clinical management [25]. PROMs represent the gold standard for spine surgery since they do not focus on the device’s technical effectiveness and safety but instead evaluate clinical outcomes regarding disability, pain, and quality-of-life improvement [11, 26]. The systematic collection of patient safety data and PROMs makes it possible to monitor surgical procedure impact and value and to research factors that might influence the outcomes and complication rates of different techniques [27]. Strömqvist et al. [16] reported that the spine registry in Sweden positively affected healthcare. It significantly reduced the national mean length of stay for microdiscectomy by maintaining the same clinical outcomes assessed using PROMs and considerably lowering the cost of the national health system. The clinical outcome of spine surgery is jeopardised by a high complication rate [28] and a narrow range of clinical improvement [29, 30]. It is based mainly on patient-reported outcome measures (PROMs) [31]. However, some procedures, such as spinal fusion surgery, are increasingly being used despite growing caution that high-quality studies are needed to support its clinical effectiveness and efficiency [32]. All the selected registries assess outcomes using different PROMs, but all consider the main domains of pain, function, and health-related quality of life (HRQoL). These outcomes are the basis on which the scientific evidence for spine registries is built [33]. Our scoping review showed that, over the years, a progressive alignment with International Consortium for Health OutcomesMeasurement (ICHOM) standards [34] has been reached, even if all the selected registries have yet to match those criteria fully.

### Registry organisation and data collection characteristics

The nine selected registries differ in structural and organisational characteristics and follow different modalities of data collection and outcome evaluation (Tables 2 and 3). As for the source of funding, many registries are funded and managed by clinician associations and receive public funding in a more or less stable way. For every case, SIRIS Spine gets a specific fee from hospitals, part of which is used to run the registry. Only the Australian and Canadian registries declare that they receive funds directly from industries. In these times of economic difficulties for many public health systems, this is good news, as it shows how spine device manufacturers are also interested in registry data. Following the introduction of the new European Medical Device Regulation (EU MDR) [35], this interest is rising, as registries can provide valuable data to accomplish post-market analysis requirements or device surveillance and traceability, assuming that rules for managing conflicts of interest are in place.

Eight of the nine selected registries have a website, but only four publish it in English. Seven of the nine selected registries publish a report, but only four are available in English. The lack of availability of information in English might represent an additional obstacle to the scientific dissemination of data. This problem might be overcome by making the information available in English by including a dedicated section on the website and by publishing reports or summaries in this language.

Seven of the nine registries analyse data by taking the number of enrolled patients as a statistical unit. Three of them also consider the total number of collected and monitored procedures. While this information is usually available in the reports, it is also published on the website in only some cases (Swespine and SIRIS Spine). There is high heterogeneity in how the data are presented and the patients/procedures are analysed. A common standard for the presentation of results is needed for readers to be able to find the information they seek. A first step to overcome this difficulty could be to define a standard form to present the principal data characterising the registry in English. An excellent example of this approach could be what has been done recently by the International Society of Arthroplasty Registries (ISAR), which has defined a form to collect such information for joint registries, sent it to all its members, and recommended putting it at the beginning of their reports or on their websites (Kajsa Erikson, ISAR administrator, personal communication to Marina Torre, email dated 01/06/2023).

Each of the selected registries collects personal, comorbidity, and diagnosis data. Two registries (German and Finnish) consider all the spinal interventions, while the others set enrolment criteria for specific anatomical districts, focusing only on some spinal disorders. Although most are based on the Glassman classification [36] or subsequent modifications, the classification systems are only sometimes declared. The results clearly show the need for a further joint classification effort, starting from the commonly used Glassman classification, which can partially catch the wide variety of clinical conditions and therapeutic approaches encountered in spine surgery. Indeed, the use of well-structured registries with data of high validity and representativeness allows for results that can be generalised and have an acceptable level of evidence, thus improving the quality and cost-effectiveness of care [37, 38]. Moreover, data collection in surgical practice allows for the documentation of pathologies and surgical approaches and the evaluation of the population in a standardised manner, thus creating a common language for benchmarking [39].

The selected registries markedly differ in their duration of follow-up. Most stop collecting data 1 or 2 years after surgery, while CSORN for over 5 years and DaneSpine, Swespine, FinSpine for over 10 years. 

Coverage and completeness are critical issues. Indeed, this information was unavailable in the published papers and needed to be requested during the survey. However, even surveys sent to the coordinators were sometimes returned with data missing. The proportion of departments involved out of the total number of departments performing spine surgery at a national level (i.e. coverage) is the first challenge: this data can easily be reached if spinal surgery centres are involved in data collection on a mandatory basis, as voluntary participation usually leads to low rates of coverage and completeness. Making data collection mandatory by law or linking data entry to reimbursements might overcome these issues [40, 41]. Coverage data are available for only six registries; among them, the Swedish and Norwegian ones show the best recruitment performance, reaching peaks of 100%. Six of the nine registries declared the completeness rate, which ranged from 3 to 86%. However, it still needs to be clarified whether all the registries managed data requests with a similar approach and whether the completeness data refer to only the registered procedures or to the follow-up as well. A completeness of 75% and over at follow-up is a reasonable threshold for accurate analyses [38, 42]. Completeness remains crucial when interpreting data from the registries and should be clearly stated in all related publications [11].

### Registry data management and quality

Spine registry data are gaining interest from all stakeholders in health systems. Indeed, the monitoring of the quality of a product used in spine surgery should be based on high-quality data, as required by the new European legislation [35]. Furthermore, these data are expected to become decisive in clinical decisions. For this reason, data entered into the registries must have a high level of completeness and accuracy. External registry monitoring would be helpful to certify the quality of the data; hence, the importance of considering systems in the registry design that monitor data quality and guarantee the accuracy of data entry. Prospective data collection, clinical data entry, quality control and data validation, study designs that control for confounding bias, and the coverage of the population enrolled and exposed to the surgery are well-defined features of an efficient registry [15].

Based on the results of our study, eight of the nine active national registries declared that they have implemented a quality control process based, for most of them, on their internal systems. None declared that they refer to external audit agencies.

### Limitations

Several difficulties emerged during our study to identify active registries in a national setting. The scoping review selected studies investigating spine surgery in a spine registry setting, though, in some cases, the authors did not cite the name of the registry on which their studies were based. In these cases, the paper was excluded to avoid bias due to misclassification, although this might have led to underestimating the real scientific impact of those registries. The same limitation applies to the possible alignment of the registries with ICHOM  standards. An intrinsic limit of our search strategy, which was based on Van Hooff et al.’s search strategy [11], is that it could only detect some of the existing active national registries because the inclusion/exclusion criteria were stringent; moreover, the original online search was performed 2 years before the analysis of results was completed. Therefore, the research process involved performing additional web searches and expert interviews to collect detailed and updated information. Finally, the aim of this study was limited to the collection of the organisational standards of the existing national registries, thus excluding the analysis of data collected in terms of their quality and comprehensiveness. Further studies might focus on this topic.

## Conclusion

Our study identified nine currently active spine surgery registries with a national setting. Overall, even though most provide substantial scientific value in terms of improving the quality of care, we found no globally accepted standards for developing a national registry of vertebral surgery. However, their experience can be a source of inspiration for other countries willing to establish a national registry and future international collaborations. An international effort to increase the comparability of the results from different registries, similar to what has been achieved by arthroplasty registries with the ISAR [43], is highly advisable.

As with all the other types of implants, spine implants must be monitored to promote evidence-based selection so that patients undergoing spine surgery will get the best and safest implants. The ODEP is an independent panel of experts providing objective ratings of the strength of evidence available on the performance of medical implants [44]. The ODEP is now increasingly recognised as a reliable source of information for surgeons, patients and hospitals about joint replacement implants. Compared to other anatomical districts, the declared methodology for spine implants published on the ODEP website [45] still needs to be settled. Reliable and internationally standardised data from national spine registries will be crucial in the following years. The ODEP’s initiative to organise the 1st International Meeting of Spinal Registries might meet these needs and pave the way to establishing a fruitful international collaboration. We hope this study will contribute to this aim and be a reference for other spine research groups, health professionals and policy-makers, as it has been for our Italian Spine Registry project.

### Supplementary Information

**Additional file 1. **Search strategy; List S1: papers included from the online database search (62); List S2: papers included from the web hand search (46).

## Data Availability

Data sharing is not applicable to this article as no datasets were generated or analysed during the current study.

## Abbreviations

BASS: British Association of Spine Surgeons
BP: Back pain after decompression
BPI: Brief Pain Inventory
BSR: British Spine Registry
CAPPS: Calgary Postoperative Pain After Spine Surgery
CD: Cervical degenerative
COMI: Core Outcome Measure Index
CSORN: Canadian Spine Outcomes and Research Network
CSREF: Canadian Spine Education and Research Fund
CSS: Canadian Spine Society
D: Discectomy
DaneSpine: Danish Spine Registry
DASS-21: Depression Anxiety Stress Scales Short Version
DDD: Degenerative disc disease
DE: Deformity
DH: Disc herniation
DI: Discitis
DS: Degenerative spondylolisthesis
DSSR: Dutch Spine Registry
DWG: German Spine Registry
EQ5D: EuroQol-5D
EQ-5D: EuroQol five-dimension scale
EQ-5D-3L: EuroQol five-dimension scale for three levels
EQ-VAS: EuroQol Visual Analogue Scale
EUMDR: European Medical Device Regulation
FABQ: Fear-Avoidance Beliefs Questionnaire
FinSpine: Finnish Registry
GA: Granuloma annulare
GPE: Global Perceived Effect instrument
RHF: Regionalt helseføretak (Regional Health Authority)
HRQOL: Health-related quality of life
HTI: Heparin-induced thrombocytopenia
ICHOM: International Consortium for Health Outcomes Measurement
IS: Idiopathic scoliosis
ISAR: International Society of Arthroplasty Registries
JOA: Japanese Orthopaedic Association
K: Kyphoplasties
LANSS: Leeds Assessment of Neuropathic Symptoms and Signs
LD: Lumbar degenerative
MCS: Multiple chemical sensitivity
MDI: Myelopathy Disability Index
mJOA: Modified Japanese Orthopaedic Association
MSPQ: Modified Somatic Perception Questionnaire
N(2)qod: National Neurosurgery Quality and Outcomes Database
NA: Not applicable
NDI: Neck Disability Index
NORspine: Norwegian Registry for Spine Surgery
NQRA: National Quality Registry Association
NRS: Numeric Rating Scale
NSQIP: National Surgical Quality Improvement Program
ODEP: Orthopaedic Data Evaluation Panel
ODI: Oswestry Disability Index
PCS: Pelvic Congestion Syndrome
PGIC: Patients' Global Impression of Change
PHQ2: Patient Health Questionnaire-2
PHQ9: Patient Health Questionnaire-9
PRISMA: Preferred Reporting Items for Systematic Reviews and Meta-Analyses
PROMs: Patient-reported outcome measures
PSAS: Translation of the Patient Scar Assessment Scale
PSFS: Patient-Specific Functional Scale
RCT: Randomised controlled trial
RE: Revisions
RIDIS: Registro Italiano Dispositivi Impiantabili per chirurgia Spinale (Italian Spine Registry)
RMDQ: Roland–Morris Disability Questionnaire
RO: Roland–Morris Disability
SBI: Pain Symptom Borthersomeness Index
SD: Spondylitis
SDR: Second Diagnosis (registered)
SF12: 12-Item Short Form Health Survey
SF-36: 36-Item Short Form Health Survey
SFI: Spine Functional Index
SI: Spinal infection
SIRIS Spine: Swiss National Implant Registry, Spine
SL: Spondylolisthesis
SP: Spondylosis
SPL: Spondylodiscitis
SPO: Spondylodeses
SRS-22r: Scoliosis Research Society 22r instrument
SS: Spinal stenosis
SSA: Spine Society of Australia
SSS: Swiss Stenosis Score
ST: Spinal tumours (primary and/or metastatic)
STR: Spinal trauma
Swespine: Swedish Spine Registry
SWISSspine: Swiss Spine Registry
UNN: Universitetssykehuset Nord-Norge (University of Northern Norway)
V: Vertebroplasty
VAS: Visual Analogue Scale for Pain
ZSDS: Zung Self-Rating Depression Scale
