# Supplementary material for: National spine surgery registries’ characteristics and aims: globally accepted standards have yet to be met. Results of a scoping review and a complementary survey
Source: J Orthop Traumatol. 2023 Sep 16;24:49. doi: 10.1186/s10195-023-00732-4 (PMC10505129; doi:10.1186/s10195-023-00732-4)
Supplement: Supplementary file 1 — Additional file 1. Search strategy; List S1: papers included from the online database search (62); List S2: papers included from the web hand search (46). [file 10195_2023_732_MOESM1_ESM.docx]

**Additional file 1**

**Search strategy**

PubMed

(“Lumbar vertebrae” [mh] OR “Spine” [mh] OR “intervertebral disk” [tiab] OR “intervertebral disc” [mh] OR spine [tiab] OR spinal [tiab] OR vertebra* [tiab] OR disc [tiab] OR discs [tiab] OR disk [tiab] OR disks [tiab] OR "sacrum" [mh] OR "epidural space" [mh] OR coccygeal [tiab] OR "dura mater" [mh] OR "dura mater" [tiab] OR "vertebral body" [mesh] OR "vertebral body" [tiab])

AND

(“low back pain ” [tiab] OR “back pain” [mh] OR “back pain” [tiab] OR “intermittent neurogenic claudication” [tiab] OR “intermittent claudication” [mh] OR “intermittent claudication” [tiab] OR ”neurogenic claudication” [tiab] OR dorsalgia [tiab] OR backache [tiab] OR lumbago [tiab] OR (lumbar [tiab] AND ("pain” [mh] OR pain [all fields])) OR “sciatica” [mh] OR sciatica [all fields] OR “spondylolisthesis” [mh] OR “spondylolisthesis” [tiab] OR “isthmic” [tiab] OR “lytic” [tiab] OR “low-grade” [tiab] OR “lumbar stenosis” [tiab] OR “spinal stenosis” [mh] OR “spinal stenosis”[tiab] OR stenosis [tiab] OR "intervertebral disc displacement" [mh] OR “intervertebral disk displacement” [tiab] OR “discitis” [mh] OR discitis [tiab] OR spondylosis [tiab] OR ((disc[tiab] OR discs[tiab] OR disk[tiab] OR disks[tiab]) AND degeneration [tiab]) OR herniated [tiab] OR hernia [tiab] OR "failed back surgery syndrome” [tiab] OR FBSS [tiab] OR myelomeningocele [tiab] OR “ankylosing spondylitis” [tiab] OR tumor [tiab] OR tumors [tiab] OR tumour [tiab] OR tumours [tiab] OR cancer [tiab] OR neoplasm [tiab] OR neoplasms [tiab] OR neoplastic [tiab] OR neoplasia [tiab] OR metastasis [tiab] OR metastases [tiab] OR metastatic [tiab] OR metastatized [tiab] OR metastatised [tiab] OR trauma [tiab] OR traumatic [tiab] OR fracture [tiab] OR "osteoarthritis, spine" [mh] OR "rigid spine syndrome" [tiab] OR "spinal dysraphism" [mh] OR "neural tube defects" [mh] OR "spinal osteochondrosis" [mh] OR "spinal curvatures" [mh] OR "spinal curvature" [tiab] OR kyphosis [tiab] OR "scheuermann disease" [tiab] OR lordosis [tiab] OR scoliosis [tiab] OR "ossification of posterior longitudinal ligament" [tiab] OR "transverse myelitis" [tiab] OR "myelitis, transverse" [mh] OR "demyelinating autoimmune diseases, CNS " [mh] OR "traumatic myelopathy"[tiab])

AND

("population register" [tiab] OR "population registers" [tiab] OR register [tiab] OR registers [tiab] OR registry [tiab] OR registries [mh])

AND

(national [tiab] OR nationally [tiab] OR country [tiab] OR state [tiab] OR public [tiab] OR federal [tiab] OR governmental [tiab] OR civic [tiab] OR civil [tiab] OR domestic [tiab] OR native [tiab] OR nationwide [tiab] OR countrywide [tiab] OR coast-to-coast[tiab] OR comprehensive [tiab] OR citizens [tiab] OR resident [tiab] OR residents)

**List S1: papers included from the online database search (62)**

1. Aghayev, E., Baerlocher, C. B., Hasdemir, M., Steinsiepe, K. F., Porchet, F., Hausmann, O., Ramadan, A. S., Maestretti, G. I., Neukamp, M., & Roder, C. P. (2012). Five-year results of cervical disc prostheses in the swissspine registry. Spine Journal, 12(9), 98S. https://doi.org/10.1016/j.spinee.2012.08.272 LK - http://QT8BH6HW4W.search.serialssolutions.com?sid=EMBASE&issn=15299430&id=doi:10.1016%2Fj.spinee.2012.08.272&atitle=Five-year+results+of+cervical+disc+prostheses+in+the+swissspine+registry&stitle=Spine+J.&title=Spine+Journal&volume=12&issue=9&spage=98S&epage=&aulast=Aghayev&aufirst=Emin&auinit=E.&aufull=Aghayev+E.&coden=&isbn=&pages=98S-&date=2012&auinit1=E&auinitm=
2. Aghayev, E., Etter, C., Bärlocher, C., Sgier, F., Otten, P., Heini, P., Hausmann, O., Maestretti, G., Baur, M., Porchet, F., Markwalder, T. M., Schären, S., Neukamp, M., & Röder, C. (2014). Five-year results of lumbar disc prostheses in the SWISSspine registry. European Spine Journal : Official Publication of the European Spine Society, the European Spinal Deformity Society, and the European Section of the Cervical Spine Research Society, 23(10), 2114–2126. https://doi.org/10.1007/s00586-014-3418-4
3. Aghayev, E., Röder, C., Zweig, T., Etter, C., & Schwarzenbach, O. (2010). Benchmarking in the SWISSspine registry: results of 52 Dynardi lumbar total disc replacements compared with the data pool of 431 other lumbar disc prostheses. European Spine Journal : Official Publication of the European Spine Society, the European Spinal Deformity Society, and the European Section of the Cervical Spine Research Society, 19(12), 2190–2199. https://doi.org/10.1007/s00586-010-1550-3
4. Ailon, T., Tee, J., Manson, N., Hall, H., Thomas, K., Rampersaud, Y. R., Yee, A., Dea, N., Glennie, A., Bailey, C., Christie, S., Weber, M. H., Nataraj, A., Paquet, J., Johnson, M., Norton, J., Ahn, H., McIntosh, G., & Fisher, C. G. (2019). Patient-reported outcomes following surgery for degenerative spondylolitshtesis: comparison of a universal and multitier health care system. The Spine Journal : Official Journal of the North American Spine Society, 19(1), 24–33. https://doi.org/10.1016/j.spinee.2018.10.005
5. Andersen Mikkel Øand Ernst, C., Rasmussen, J., Ankjær, T., & Carreon, L. Y. (2020). Predictive Factors of Successful Return to Work Following Discectomy. Global Spine Journal, 2192568220960399. https://doi.org/10.1177/2192568220960399
6. Andersen, M. Ø., Fritzell, P., Eiskjaer, S. P., Lagerbäck, T., Hägg, O., Nordvall, D., Lönne, G., Solberg, T., Jacobs, W., van Hooff, M., Gerdhem, P., & Gehrchen, M. (2019). Surgical Treatment of Degenerative Disk Disease in Three Scandinavian Countries: An International Register Study Based on Three Merged National Spine Registers. Global Spine Journal, 9(8), 850–858. https://doi.org/10.1177/2192568219838535
7. Austevoll, I. M., Gjestad, R., Grotle, M., Solberg, T., Brox, J. I., Hermansen, E., Rekeland, F., Indrekvam, K., Storheim, K., & Hellum, C. (2019). Follow-up score, change score or percentage change score for determining clinical important outcome following surgery? An observational study from the Norwegian registry for Spine surgery evaluating patient reported outcome measures in lumbar spinal sten. BMC Musculoskeletal Disorders, 20(1), 31. https://doi.org/10.1186/s12891-018-2386-y
8. Austevoll, I. M., Gjestad, R., Solberg, T., Storheim, K., Brox, J. I., Hermansen, E., Rekeland, F., Indrekvam, K., & Hellum, C. (2020). Comparative Effectiveness of Microdecompression Alone vs Decompression Plus Instrumented Fusion in Lumbar Degenerative Spondylolisthesis. JAMA Network Open, 3(9), e2015015. https://doi.org/10.1001/jamanetworkopen.2020.15015
9. Beck, J., Westin, O., Klingenstierna, M., & Baranto, A. (2020). Successful Introduction of Full-Endoscopic Lumbar Interlaminar Discectomy in Sweden. International Journal of Spine Surgery, 14(4), 563–570. https://doi.org/10.14444/7075
10. Breakwell, L. M., Cole, A. A., Birch, N., & Heywood, C. (2015). Should we all go to the PROM? The first two years of the British Spine Registry. In The bone {\&} joint journal (Vols. 97-B, Issue 7, pp. 871–874). https://doi.org/10.1302/0301-620X.97B7.35391
11. Canizares, M., Gleenie, R. A., Perruccio, A. V, Abraham, E., Ahn, H., Attabib, N., Christie, S., Johnson, M. G., Nataraj, A., Nicholls, F., Paquet, J., Phan, P., Rasoulinejad, P., Manson, N., Hall, H., Thomas, K., Fisher, C. G., & Rampersaud, Y. R. (2020). Patients’ expectations of spine surgery for degenerative conditions: results from the Canadian Spine Outcomes and Research Network (CSORN). The Spine Journal : Official Journal of the North American Spine Society, 20(3), 399–408. https://doi.org/10.1016/j.spinee.2019.10.001
12. Comins, J., Brodersen, J., Wedderkopp, N., Lassen, M. R., Shakir, H., Specht, K., Brorson, S., & Christensen, K. B. (2020). Psychometric Validation of the Danish Version of the Oswestry Disability Index in Patients With Chronic Low Back Pain. Spine, 45(16), 1143–1150. https://doi.org/10.1097/BRS.0000000000003486
13. Diel, P., Reuss, W., Aghayev, E., Moulin, P., & Röder, C. (2010). SWISSspine-a nationwide health technology assessment registry for balloon kyphoplasty: methodology and first results. The Spine Journal : Official Journal of the North American Spine Society, 10(11), 961–971. https://doi.org/10.1016/j.spinee.2009.08.452
14. Divecha, H. M., Siddique, I., Breakwell, L. M., & Millner, P. A. (2014). Complications in spinal deformity surgery in the United Kingdom: 5-year results of the annual British Scoliosis Society National Audit of Morbidity and Mortality. European Spine Journal : Official Publication of the European Spine Society, the European Spinal Deformity Society, and the European Section of the Cervical Spine Research Society, 23 Suppl 1(Suppl 1), S55--60. https://doi.org/10.1007/s00586-014-3197-y
15. Elkan, P., Lagerbäck, T., Möller, H., & Gerdhem, P. (2018). Response rate does not affect patient-reported outcome after lumbar discectomy. European Spine Journal : Official Publication of the European Spine Society, the European Spinal Deformity Society, and the European Section of the Cervical Spine Research Society, 27(7), 1538–1546. https://doi.org/10.1007/s00586-018-5541-0
16. Fors, M., Enthoven, P., Abbott, A., & Öberg, B. (2019). Effects of pre-surgery physiotherapy on walking ability and lower extremity strength in patients with degenerative lumbar spine disorder: Secondary outcomes of the PREPARE randomised controlled trial. BMC Musculoskeletal Disorders, 20(1), 468. https://doi.org/10.1186/s12891-019-2850-3
17. Försth, P., Michaëlsson, K., Sandén, B., Forsth, P., Michaelsson, K., & Sanden, B. (2013). Does fusion improve the outcome after decompressive surgery for lumbar spinal stenosis?: A TWO-YEAR FOLLOW-UP STUDY INVOLVING 5390 PATIENTS. The Bone {\&} Joint Journal, 95-B(7), 980–985. https://doi.org/10.1302/0301-620X.95B7.30776
18. Fritzell, P., Stromqvist, B., & Hagg, O. (2006). A practical approach to spine registers in {\{}Europe{\}}: the {\{}Swedish{\}} experience. European Spine Journal, 15, S57----S63. https://doi.org/10.1007/s00586-005-1051-y
19. Germon, T., Ahuja, S., Casey, A. T. H., Todd, N. V, & Rai, A. (2015). British Association of Spine Surgeons standards of care for cauda equina syndrome. In The spine journal : official journal of the North American Spine Society (Vol. 15, Issue 3 Suppl, pp. S2--S4). https://doi.org/10.1016/j.spinee.2015.01.006
20. Grotle, M., Solberg, T., Storheim, K., Lærum, E., & Zwart, J.-A. (2014). Public and private health service in Norway: a comparison of patient characteristics and surgery criteria for patients with nerve root affections due to discus herniation. European Spine Journal : Official Publication of the European Spine Society, the European Spinal Deformity Society, and the European Section of the Cervical Spine Research Society, 23(9), 1984–1991. https://doi.org/10.1007/s00586-014-3293-z
21. Hareni, N., Strömqvist, F., Strömqvist, B., Rosengren, B. E., & Karlsson, M. K. (2019). Predictors of satisfaction after lumbar disc herniation surgery in elderly. BMC Musculoskeletal Disorders, 20(1), 594. https://doi.org/10.1186/s12891-019-2975-4
22. Hermansen, E., Myklebust, T. Å., Austevoll, I. M., Rekeland, F., Solberg, T., Storheim, K., Grundnes, O., Aaen, J., Brox, J. I., Hellum, C., & Indrekvam, K. (2019). Clinical outcome after surgery for lumbar spinal stenosis in patients with insignificant lower extremity pain. A prospective cohort study from the Norwegian registry for spine surgery. BMC Musculoskeletal Disorders, 20(1), 36. https://doi.org/10.1186/s12891-019-2407-5
23. Hübschle, L., Borgström, F., Olafsson, G., Röder, C., Moulin, P., Popp, A. W., Külling, F., Aghayev, E., Hubschle, L., Borgstrom, F., Olafsson, G., Roder, C., Moulin, P., Popp, A. W., Kulling, F., Aghayev, E., Hübschle, L., Borgström, F., Olafsson, G., … Aghayev, E. (2014). Real-life results of balloon kyphoplasty for vertebral compression fractures from the {\{}SWISSspine{\}} registry. Spine Journal, 14(9), 2063–2077. https://doi.org/10.1016/j.spinee.2013.12.019
24. Iderberg, H., Willers, C., Borgström, F., Hedlund, R., Hägg, O., Möller, H., Ornstein, E., Sandén, B., Stalberg, H., Torevall-Larsson, H., Tullberg, T., & Fritzell, P. (2019). Predicting clinical outcome and length of sick leave after surgery for lumbar spinal stenosis in Sweden: a multi-register evaluation. European Spine Journal : Official Publication of the European Spine Society, the European Spinal Deformity Society, and the European Section of the Cervical Spine Research Society, 28(6), 1423–1432. https://doi.org/10.1007/s00586-018-5842-3
25. Jansson, K.-A., Németh, G., Granath, F., Jönsson, B., & Blomqvist, P. (2005). Health-related quality of life in patients before and after surgery for a herniated lumbar disc. The Journal of Bone and Joint Surgery. British Volume, 87(7), 959–964. https://doi.org/10.1302/0301-620X.87B7.16240
26. Jansson, K.-A., Németh, G., Granath, F., Jönsson, B., & Blomqvist, P. (2009). Health-related quality of life (EQ-5D) before and one year after surgery for lumbar spinal stenosis. The Journal of Bone and Joint Surgery. British Volume, 91(2), 210–216. https://doi.org/10.1302/0301-620X.91B2.21119
27. Joelson, A., Nerelius, F., Holy, M., & Sigmundsson, F. G. (n.d.). Reoperations after decompression with or without fusion for {\{}L4{\}}-5 spinal stenosis with or without degenerative spondylolisthesis: a study of 6,532 patients in {\{}Swespine{\}}, the national {\{}Swedish{\}} spine register. Acta Orthopaedica. https://doi.org/10.1080/17453674.2021.1879505
28. Jonsson, E., Hansson-Hedblom, A., Kirketeig, T., Fritzell, P., Hägg, O., & Borgström, F. (2020). Cost and Health Outcomes Patterns in Patients Treated With Spinal Cord Stimulation Following Spine Surgery-A Register-Based Study. Neuromodulation : Journal of the International Neuromodulation Society, 23(5), 626–633. https://doi.org/10.1111/ner.13056
29. Jonsson, E., Olafsson, G., Fritzell, P., Hägg, O., & Borgström, F. (2015). Productivity loss due to low back pain: Results from swedish registers. Value in Health, 18(7), A649. https://www.embase.com/search/results?subaction=viewrecord%7B%5C&%7Did=L72084586%7B%5C&%7Dfrom=export
30. Jonsson, E., Olafsson, G., Fritzell, P., Hägg, O., & Borgström, F. (2017). Cost of low back pain: Results from a register study in Sweden. European Spine Journal, 26(2), S404--S405. https://doi.org/10.1007/s00586-017-5225-1 LK - http://QT8BH6HW4W.search.serialssolutions.com?sid=EMBASE&issn=14320932&id=doi:10.1007%2Fs00586-017-5225-1&atitle=Cost+of+low+back+pain%3A+Results+from+a+register+study+in+Sweden&stitle=Eur.+Spine+J.&title=European+Spine+Journal&volume=26&issue=2&spage=S404&epage=S405&aulast=Jonsson&aufirst=Emma&auinit=E.&aufull=Jonsson+E.&coden=&isbn=&pages=S404-S405&date=2017&auinit1=E&auinitm=
31. Lagerbäck, T., Elkan, P., Möller, H., Grauers, A., Diarbakerli, E., Gerdhem, P., Lagerback, T., Elkan, P., Moller, H., Grauers, A., Diarbakerli, E., & Gerdhem, P. (2015). An observational study on the outcome after surgery for lumbar disc herniation in adolescents compared with adults based on the Swedish Spine Register. The Spine Journal : Official Journal of the North American Spine Society, 15(6), 1241–1247. https://doi.org/10.1016/j.spinee.2015.02.024
32. Lagerback, T., Fritzell, P., Hagg, O., Nordvall, D., Lonne, G., Solberg, T. K., Andersen Mikkel O Øand Eiskjaer, S., Gehrchen, M., Jacobs, W. C., van Hooff, M. L., Gerdhem, P., Lagerbäck, T., Fritzell, P., Hägg, O., Nordvall, D., Lønne, G., Solberg, T. K., Andersen Mikkel O Øand Eiskjær, S., Gehrchen, M., … Gerdhem, P. (2019). Effectiveness of surgery for sciatica with disc herniation is not substantially affected by differences in surgical incidences among three countries: results from the {\{}Danish{\}}, {\{}Swedish{\}} and {\{}Norwegian{\}} spine registries. European Spine Journal, 28(11), 2562–2571. https://doi.org/10.1007/s00586-018-5768-9
33. Lagerback, T., Moller, H., & Gerdhem, P. (2019). Lumbar disc herniation surgery in adolescents and young adults {\{}LONG{\}}-{\{}TERM{\}} {\{}OUTCOME{\}} {\{}COMPARISON{\}}. Bone {\&} Joint Journal, 101B(12), 1534–1541. https://doi.org/10.1302/0301-620X.101B12.BJJ-2019-0621.R1
34. Lonne, G., Fritzell, P., Hagg, O., Nordvall, D., Gerdhem, P., Lagerback, T., Andersen, M., Eiskjaer, S. S., Gehrchen, M., Jacobs, W., van Hooff, M. L., Solberg, T. K., Lønne, G., Fritzell, P., Hägg, O., Nordvall, D., Gerdhem, P., Lagerbäck, T., Andersen, M., … Solberg, T. K. (2019). Lumbar spinal stenosis: comparison of surgical practice variation and clinical outcome in three national spine registries. The Spine Journal : Official Journal of the North American Spine Society, 19(1), 41–49. https://doi.org/10.1016/j.spinee.2018.05.028
35. Lonne, G., Schoenfeld, A. J., Cha, T. D., Nygaard, O. P., Zwart, J. A. H., & Solberg, T. (2017). Variation in selection criteria and approaches to surgery for {\{}Lumbar{\}} {\{}Spinal{\}} {\{}Stenosis{\}} among patients treated in {\{}Boston{\}} and {\{}Norway{\}}. Clinical Neurology and Neurosurgery, 156, 77–82. https://doi.org/10.1016/j.clineuro.2017.03.008
36. MacDowall, A., Heary, R. F., Holy, M., Lindhagen, L., & Olerud, C. (2020). Posterior foraminotomy versus anterior decompression and fusion in patients with cervical degenerative disc disease with radiculopathy: up to 5 years of outcome from the national {\{}Swedish{\}} {\{}Spine{\}} {\{}Register{\}}. Journal of Neurosurgery-Spine, 32(3), 344–352. https://doi.org/10.3171/2019.9.SPINE19787
37. MacDowall, A., Skeppholm, M., Lindhagen, L., Robinson, Y., Löfgren, H., Michaëlsson, K., Olerud, C., Lofgren, H., Michaelsson, K., Olerud, C., Löfgren, H., Michaëlsson, K., & Olerud, C. (2018). Artificial disc replacement versus fusion in patients with cervical degenerative disc disease with radiculopathy: 5-year outcomes from the National Swedish Spine Register. Journal of Neurosurgery. Spine, 30(2), 159–167. https://doi.org/10.3171/2018.7.SPINE18657
38. Ohrn, A., Olai, A., Rutberg, H., Nilsen, P., & Tropp, H. (2011). Adverse events in spine surgery in Sweden: a comparison of patient claims data and national quality register (Swespine) data . Acta Orthopaedica, 82(6), 727–731. https://doi.org/10.3109/17453674.2011.636673
39. Parai, C., Hägg, O., Willers, C., Lind, B., & Brisby, H. (2020). Characteristics and predicted outcome of patients lost to follow-up after degenerative lumbar spine surgery. European Spine Journal : Official Publication of the European Spine Society, the European Spinal Deformity Society, and the European Section of the Cervical Spine Research Society, 29(12), 3063–3073. https://doi.org/10.1007/s00586-020-06528-y
40. Rischke, B., Zimmers, K. B., & Smith, E. (2015). Viscoelastic Disc Arthroplasty Provides Superior Back and Leg Pain Relief in Patients with Lumbar Disc Degeneration Compared to Anterior Lumbar Interbody Fusion. International Journal of Spine Surgery, 9, 26. https://doi.org/10.14444/2026
41. Röder, C., Boszczyk, B., Perler, G., Aghayev, E., Külling, F., & Maestretti, G. (2013). Cement volume is the most important modifiable predictor for pain relief in BKP: results from SWISSspine, a nationwide registry. European Spine Journal : Official Publication of the European Spine Society, the European Spinal Deformity Society, and the European Section of the Cervical Spine Research Society, 22(10), 2241–2248. https://doi.org/10.1007/s00586-013-2869-3
42. Schluessmann, E., Aghayev, E., Staub, L., Moulin, P., Zweig, T., & Röder, C. (2010). SWISSspine: the case of a governmentally required HTA-registry for total disc arthroplasty: results of cervical disc prostheses. Spine, 35(24), E1397--405. https://doi.org/10.1097/BRS.0b013e3181e0e871
43. Schluessmann, E., Diel, P., Aghayev, E., Zweig, T., Moulin, P., & Röder, C. (2009). SWISSspine: a nationwide registry for health technology assessment of lumbar disc prostheses. European Spine Journal : Official Publication of the European Spine Society, the European Spinal Deformity Society, and the European Section of the Cervical Spine Research Society, 18(6), 851–861. https://doi.org/10.1007/s00586-009-0934-8
44. Stottrup, C. C., Andresen, A. K., Carreon, L., Andersen Mikkel O ØO Øand Støttrup, C. C., Andresen, A. K., Carreon, L., Andersen Mikkel O ØO Øand Stottrup, C. C., Andresen, A. K., Carreon, L., Andersen Mikkel O ØO Øand Støttrup, C. C., Andresen, A. K., Carreon, L., & Andersen, M. O. Ø. O. Ø. (2019). Increasing reoperation rates and inferior outcome with prolonged symptom duration in lumbar disc herniation surgery - a prospective cohort study. Spine Journal, 19(9), 1463–1469. https://doi.org/10.1016/j.spinee.2019.04.001
45. Strömqvist, B. (2002). Evidence-based lumbar spine surgery. The role of national registration. Acta Orthopaedica Scandinavica. Supplementum, 73(305), 34–39. https://doi.org/10.1080/000164702760379530
46. Strömqvist, B., Fritzell, P., Hägg, O., & Jönsson, B. (2005). One-year report from the Swedish National Spine Register. Swedish Society of Spinal Surgeons. Acta Orthopaedica. Supplementum, 76(319), 1–24. https://doi.org/10.1080/17453690510041950
47. Strömqvist, B., Fritzell, P., Hägg, O., Jönsson, B., & Sandén, B. (2013). Swespine: the Swedish spine register : the 2012 report. European Spine Journal : Official Publication of the European Spine Society, the European Spinal Deformity Society, and the European Section of the Cervical Spine Research Society, 22(4), 953–974. https://doi.org/10.1007/s00586-013-2758-9
48. Strömqvist, B., Jönsson, B., Fritzell, P., Hägg, O., Larsson, B. E., & Lind, B. (2001). The Swedish National Register for lumbar spine surgery: Swedish Society for Spinal Surgery. Acta Orthopaedica Scandinavica, 72(2), 99–106. https://doi.org/10.1080/000164701317323327
49. Strömqvist, F., Sigmundsson, F. G., Strömqvist, B., Jönsson, B., Karlsson, M. K., Stromqvist, F., Sigmundsson, F. G., Stromqvist, B., Jonsson, B., & Karlsson, M. K. (2019). Incidental durotomy in degenerative lumbar spine surgery - a register study of 64,431 operations. Spine Journal, 19(4), 624–630. https://doi.org/10.1016/j.spinee.2018.08.012
50. Strömqvist, F., Strömqvist, B., & Jönsson, B. (2016). The outcome of lumbar disc herniation surgery is worse in old adults than in young adults: A study of 14,090 individuals in the Swedish Spine Surgery Register (SweSpine). Acta Orthopaedica, 87(5), 516–521. https://doi.org/10.1080/17453674.2016.1205173 LK - http://QT8BH6HW4W.search.serialssolutions.com?sid=EMBASE&issn=17453682&id=doi:10.1080%2F17453674.2016.1205173&atitle=The+outcome+of+lumbar+disc+herniation+surgery+is+worse+in+old+adults+than+in+young+adults%3A+A+study+of+14%2C090+individuals+in+the+Swedish+Spine+Surgery+Register+%28SweSpine%29&stitle=Acta+Orthop.&title=Acta+Orthopaedica&volume=87&issue=5&spage=516&epage=521&aulast=Str%C3%B6mqvist&aufirst=Fredrik&auinit=F.&aufull=Str%C3%B6mqvist+F.&coden=&isbn=&
51. Stromqvist, F., Stromqvist, B., Jonsson, B., & Karlsson, M. K. (2016). Inferior {\{}Outcome{\}} of {\{}Lumbar{\}} {\{}Disc{\}} {\{}Surgery{\}} in {\{}Women{\}} {\{}Due{\}} to {\{}Inferior{\}} {\{}Preoperative{\}} {\{}Status{\}} {\{}A{\}} {\{}Prospective{\}} {\{}Study{\}} in 11,237 {\{}Patients{\}}. Spine, 41(15), 1247–1252. https://doi.org/10.1097/BRS.0000000000001492
52. Strömqvist, F., Strömqvist, B., Jönsson, B., & Karlsson, M. K. (2016). Gender differences in patients scheduled for lumbar disc herniation surgery: a National Register Study including 15,631 operations. European Spine Journal : Official Publication of the European Spine Society, the European Spinal Deformity Society, and the European Section of the Cervical Spine Research Society, 25(1), 162–167. https://doi.org/10.1007/s00586-015-4052-5
53. Strömqvist, F., Strömqvist, B., Jönsson, B., & Karlsson, M. K. (2016). Gender differences in the surgical treatment of lumbar disc herniation in elderly. European Spine Journal : Official Publication of the European Spine Society, the European Spinal Deformity Society, and the European Section of the Cervical Spine Research Society, 25(11), 3528–3535. https://doi.org/10.1007/s00586-016-4638-6
54. Strömqvist, F., Strömqvist, B., Jönsson, B., & Karlsson, M. K. (2017). Surgical treatment of lumbar disc herniation in different ages-evaluation of 11,237 patients. The Spine Journal : Official Journal of the North American Spine Society, 17(11), 1577–1585. https://doi.org/10.1016/j.spinee.2017.03.013
55. Stromqvist, F., Stromqvist, B., Jonsson, B., Gerdhem, P., Karlsson, M. K., Strömqvist, F., Strömqvist, B., Jönsson, B., Gerdhem, P., Karlsson, M. K., Stromqvist, F., Stromqvist, B., Jonsson, B., Gerdhem, P., Karlsson, M. K., Strömqvist, F., Strömqvist, B., Jönsson, B., Gerdhem, P., & Karlsson, M. K. (2016). Predictive outcome factors in the young patient treated with lumbar disc herniation surgery. Journal of Neurosurgery-Spine, 25(4), 448–455. https://doi.org/10.3171/2016.2.SPINE16136
56. Strömqvist, F., Strömqvist, B., Jönsson, B., Gerdhem, P., Karlsson, M. K., Stromqvist, F., Stromqvist, B., Jonsson, B., Gerdhem, P., & Karlsson, M. K. (2015). Outcome of surgical treatment of lumbar disc herniation in young individuals. The Bone {\&} Joint Journal, 97-B(12), 1675–1682. https://doi.org/10.1302/0301-620X.97B12.36258
57. Triebel, J., Snellman, G., Sandén, B., Strömqvist, F., & Robinson, Y. (2017). Women do not fare worse than men after lumbar fusion surgery: Two-year follow-up results from 4,780 prospectively collected patients in the Swedish National Spine Register with lumbar degenerative disc disease and chronic low back pain. The Spine Journal : Official Journal of the North American Spine Society, 17(5), 656–662. https://doi.org/10.1016/j.spinee.2016.11.001
58. Udby, P. M., Ohrt-Nissen, S., Bendix, T., Paulsen, R., Støttrup, C., Andresen, A., Brorson, S., Carreon, L. Y., & Andersen, M. Ø. (2020). Are Modic Changes Associated With Health-related Quality of Life After Discectomy: A Study on 620 Patients With Two-year Follow-up. Spine, 45(21), 1491–1497. <https://doi.org/10.1097/BRS.0000000000003618>
59. Vinas-Rios, J. M., Rauschmann, M., Sellei, R., Arabmotlagh, M., Sobotke, R., & Meyer, F. (2020). Intra-and postoperative complications in the treatment of spinal metastases. A multicentre surveillance study from the German Spinal Registry (DWG-Register). Journal of Neurosurgical Sciences. https://doi.org/10.23736/S0390-5616.20.05029-8
60. Zanoli, G., Strömqvist, B., & Jönsson, B. (2001). Visual analog scales for interpretation of back and leg pain intensity in patients operated for degenerative lumbar spine disorders. Spine, 26(21), 2375–2380. https://doi.org/10.1097/00007632-200111010-00015
61. Zweig, T., Aghayev, E., Melloh, M., Dietrich, D., & Röder, C. (2012). Influence of preoperative leg pain and radiculopathy on outcomes in mono-segmental lumbar total disc replacement: results from a nationwide registry. European Spine Journal : Official Publication of the European Spine Society, the European Spinal Deformity Society, and the European Section of the Cervical Spine Research Society, 21 Suppl 6(Suppl 6), S729--36. https://doi.org/10.1007/s00586-011-1863-x
62. Zweig, T., Hemmeler, C., Aghayev, E., Melloh, M., Etter, C., & Röder, C. (2011). Influence of preoperative nucleus pulposus status and radiculopathy on outcomes in mono-segmental lumbar total disc replacement: results from a nationwide registry. BMC Musculoskeletal Disorders, 12, 275. https://doi.org/10.1186/1471-2474-12-275

**List S2: papers included from the web hand search (46)**

1. Aghayev, E., Henning, J., Munting, E., Diel, P., Moulin, P., & Röder, C. (2012). Comparative effectiveness research across two spine registries. European Spine Journal : Official Publication of the European Spine Society, the  European Spinal Deformity Society, and the European Section of the Cervical Spine Research Society, 21(8), 1640–1647. https://doi.org/10.1007/s00586-012-2256-5
2. Andresen, A. K., Paulsen, R. T., Busch, F., Isenberg-Jørgensen, A., Carreon, L. Y., & Andersen, M. Ø. (2018). Patient-Reported Outcomes and Patient-Reported Satisfaction After Surgical Treatment  for Cervical Radiculopathy. Global Spine Journal, 8(7), 703–708. https://doi.org/10.1177/2192568218765398
3. Beck, J., Westin, O., Brisby, H., & Baranto, A. (2021). Association of extended duration of sciatic leg pain with worse outcome after lumbar  disc herniation surgery: a register study in 6216 patients. Journal of Neurosurgery. Spine, 1–9. https://doi.org/10.3171/2020.8.SPINE20602
4. Berg, S., & Gillberg-Aronsson, N. (2015). Clinical outcomes after treatment with disc prostheses in three lumbar segments  compared to one- or two segments. International Journal of Spine Surgery, 9, 49. https://doi.org/10.14444/2049
5. Berg, S., Fritzell, P., & Tropp, H. (2009). Sex life and sexual function in men and women before and after total disc  replacement compared with posterior lumbar fusion. The Spine Journal : Official Journal of the North American Spine Society, 9(12), 987–994. https://doi.org/10.1016/j.spinee.2009.08.454
6. Covaro, A. A., Manabe, N., Bobinski, L., Olerud, C., & Robinson, Y. (2017). The importance of the occipitocervical area in patients with ankylosing spondylitis  analysis of a cohort of 86 cervical fractures in surgically treated patients. Journal of Craniovertebral Junction & Spine, 8(4), 374–377. https://doi.org/10.4103/jcvjs.JCVJS_115_17
7. Cushnie, D., Thomas, K., Jacobs, W. B., Cho, R. K. H., Soroceanu, A., Ahn, H., Attabib, N., Bailey, C. S., Fisher, C. G., Glennie, R. A., Hall, H., Jarzem, P., Johnson, M. G., Manson, N. A., Nataraj, A., Paquet, J., Rampersaud, Y. R., Phan, P., & Casha, S. (2019). Effect of preoperative symptom duration on outcome in lumbar spinal stenosis: a  Canadian Spine Outcomes and Research Network registry study. The Spine Journal : Official Journal of the North American Spine Society, 19(9), 1470–1477. https://doi.org/10.1016/j.spinee.2019.05.008
8. Eastwood, D., Manson, N., Bigney, E., Darling, M., Richardson, E., Paixao, R., Underwood, T., Ellis, K., & Abraham, E. (2019). Improving postoperative patient reported benefits and satisfaction following spinal  fusion with a single preoperative education session. The Spine Journal : Official Journal of the North American Spine Society, 19(5), 840–845. https://doi.org/10.1016/j.spinee.2018.11.010
9. Elkan, P., Sjövie Hasserius, J., & Gerdhem, P. (2016). Similar result after non-elective and elective surgery for lumbar disc herniation:  an observational study based on the SweSpine register. European Spine Journal : Official Publication of the European Spine Society, the  European Spinal Deformity Society, and the European Section of the Cervical Spine Research Society, 25(5), 1460–1466. https://doi.org/10.1007/s00586-016-4419-2
10. Ersberg, A., & Gerdhem, P. (2013). Pre- and postoperative quality of life in patients treated for scoliosis. Acta Orthopaedica, 84(6), 537–543. https://doi.org/10.3109/17453674.2013.854667
11. Evaniew, N., Cadotte, D. W., Dea, N., Bailey, C. S., Christie, S. D., Fisher, C. G., Paquet, J., Soroceanu, A., Thomas, K. C., Rampersaud, Y. R., Manson, N. A., Johnson, M., Nataraj, A., Hall, H., McIntosh, G., & Jacobs, W. B. (2020). Clinical predictors of achieving the minimal clinically important difference after  surgery for cervical spondylotic myelopathy: an external validation study from the Canadian Spine Outcomes and Research Network. Journal of Neurosurgery. Spine, 1–9. https://doi.org/10.3171/2020.2.SPINE191495
12. Försth, P., Svedmark, P., Noz, M. E., Maguire, G. Q. J., Zeleznik, M. P., & Sandén, B. (2018). Motion Analysis in Lumbar Spinal Stenosis With Degenerative Spondylolisthesis: A  Feasibility Study of the 3DCT Technique Comparing Laminectomy Versus Bilateral Laminotomy. Clinical Spine Surgery, 31(8), E397–E402. https://doi.org/10.1097/BSD.0000000000000677
13. Glassman, S., Carreon, L. Y., Andersen, M., Asher, A., Eiskjær, S., Gehrchen, M., Imagama, S., Ishii, K., Kaito, T., Matsuyama, Y., Moridaira, H., Mummaneni, P., Shaffrey, C., & Matsumoto, M. (2017). Predictors of Hospital Readmission and Surgical Site Infection in the United States,  Denmark, and Japan: Is Risk Stratification a Universal Language? Spine, 42(17), 1311–1315. https://doi.org/10.1097/BRS.0000000000002082
14. Gulati, S., Nordseth, T., Nerland, U. S., Gulati, M., Weber, C., Giannadakis, C., Nygaard, Ø. P., Solberg, T. K., Solheim, O., & Jakola, A. S. (2015). Does daily tobacco smoking affect outcomes after microdecompression for degenerative  central lumbar spinal stenosis? - A multicenter observational registry-based study. Acta Neurochirurgica, 157(7), 1157–1164. https://doi.org/10.1007/s00701-015-2437-1
15. Hansson-Hedblom, A., Jonsson, E., Fritzell, P., Hägg, O., & Borgström, F. (2019). The Association Between Patient Reported Outcomes of Spinal Surgery and Societal  Costs: A Register Based Study. Spine, 44(18), 1309–1317. https://doi.org/10.1097/BRS.0000000000003050
16. Hareni, N., Strömqvist, F., Strömqvist, B., Sigmundsson, F. G., Rosengren, B. E., & Karlsson, M. K. (2021). Back pain is also improved by lumbar disc herniation surgery. Acta Orthopaedica, 92(1), 4–8. https://doi.org/10.1080/17453674.2020.1815981
17. Hermansen, E., Romild, U. K., Austevoll, I. M., Solberg, T., Storheim, K., Brox, J. I., Hellum, C., & Indrekvam, K. (2017). Does surgical technique influence clinical outcome after lumbar spinal stenosis  decompression? A comparative effectiveness study from the Norwegian Registry for Spine Surgery. European Spine Journal : Official Publication of the European Spine Society, the  European Spinal Deformity Society, and the European Section of the Cervical Spine Research Society, 26(2), 420–427. https://doi.org/10.1007/s00586-016-4643-9
18. Herren, C., Aghayev, E., Kaulhausen, T., Roeder, C., Meyer, F., Siewe, J., & Sobottke, R. (2014). [Influencing factors on the length of stay in lumbar spine surgery : analysis of the  German spine registry]. Der Orthopade, 43(12), 1043–1051. https://doi.org/10.1007/s00132-014-3033-4
19. Kirkegaard, A. O., Sørensen, S. T., Ziegler, D. S., Carreon, L., Andersen, M. Ø., & Rousing, R. (2018). Percutaneous vertebroplasty is safe and effective for cancer-related vertebral  compression fractures. Danish Medical Journal, 65(10).
20. Knutsson, B., Michaëlsson, K., & Sandén, B. (2014). Obese patients report modest weight loss after surgery for lumbar spinal stenosis: a  study from the Swedish spine register. Spine, 39(20), 1725–1730. https://doi.org/10.1097/BRS.0000000000000464
21. Meyer, B., Shiban, E., Albers, L. E., & Krieg, S. M. (2020). Completeness and accuracy of data in spine registries: an independent audit-based  study. European Spine Journal : Official Publication of the European Spine Society, the  European Spinal Deformity Society, and the European Section of the Cervical Spine Research Society, 29(6), 1453–1461. https://doi.org/10.1007/s00586-020-06342-6
22. Mjåset, C., Zwart, J.-A., Goedmakers, C. M. W., Smith, T. R., Solberg, T. K., & Grotle, M. (2020). Criteria for success after surgery for cervical radiculopathy-estimates for a  substantial amount of improvement in core outcome measures. The Spine Journal : Official Journal of the North American Spine Society, 20(9), 1413–1421. https://doi.org/10.1016/j.spinee.2020.05.549
23. Morcos, M. W., Jiang, F., McIntosh, G., Ahn, H., Dea, N., Abraham, E., Paquet, J., Natara, A., Johnson, M., Manson, N., Fisher, C., Rampersaud, R., Thomas, K., Hall, H., & Weber, M. (2019). Predictive Factors for Discharge Destination Following Posterior Lumbar Spinal  Fusion: A Canadian Spine Outcome and Research Network (CSORN) Study. Global Spine Journal, 9(4), 403–408. https://doi.org/10.1177/2192568218797090
24. Parai, C., Hägg, O., Lind, B., & Brisby, H. (2020). ISSLS prize in clinical science 2020: the reliability and interpretability of score  change in lumbar spine research. European Spine Journal : Official Publication of the European Spine Society, the  European Spinal Deformity Society, and the European Section of the Cervical Spine Research Society, 29(4), 663–669. https://doi.org/10.1007/s00586-019-06222-8
25. Paulsen, R. T., Bouknaitir, J. B., Fruensgaard, S., Carreron, L., & Andersen, M. (2016). Patient are satisfied one year after decompression surgery for lumbar spinal  stenosis. Danish Medical Journal, 63(11).
26. Robinson, Y., Michaëlsson, K., & Sandén, B. (2013). Instrumentation in lumbar fusion improves back pain but not quality of life 2 years  after surgery. A study of 1,310 patients with degenerative disc disease from the Swedish Spine Register SWESPINE. Acta Orthopaedica, 84(1), 7–11. https://doi.org/10.3109/17453674.2013.771300
27. Röder, C., Baumgärtner, B., Berlemann, U., & Aghayev, E. (2015). Superior outcomes of decompression with an interlaminar dynamic device versus  decompression alone in patients with lumbar spinal stenosis and back pain: a cross registry study. European Spine Journal : Official Publication of the European Spine Society, the  European Spinal Deformity Society, and the European Section of the Cervical Spine Research Society, 24(10), 2228–2235. https://doi.org/10.1007/s00586-015-4124-6
28. Romagna, A., Wilson, J. R., Jacobs, W. B., Johnson, M. G., Bailey, C. S., Christie, S., Paquet, J., Nataraj, A., Cadotte, D. W., Manson, N., Hall, H., Thomas, K. C., Schwartz, C., Rampersaud, Y. R., McIntosh, G., Fisher, C. G., & Dea, N. (2020). Factors Associated With Return to Work After Surgery for Degenerative Cervical  Spondylotic Myelopathy: Cohort Analysis From the Canadian Spine Outcomes and Research Network. Global Spine Journal, 2192568220958669. https://doi.org/10.1177/2192568220958669
29. Rowe, E., Hassan, E., Carlesso, L., Astephen Wilson, J., Gross, D. P., Fisher, C., Hall, H., Manson, N., Thomas, K., McIntosh, G., Drew, B., Rampersaud, R., & Macedo, L. (2020). Predicting recovery after lumbar spinal stenosis surgery: A protocol for a  historical cohort study using data from the Canadian Spine Outcomes Research Network (CSORN). Canadian Journal of Pain = Revue Canadienne de La Douleur, 4(4), 19–25. https://doi.org/10.1080/24740527.2020.1734918
30. Rudolfsen, J. H., Solberg, T. K., Ingebrigtsen, T., & Olsen, J. A. (2020). Associations between utilization rates and patients’ health: a study of spine  surgery and patient-reported outcomes (EQ-5D and ODI). BMC Health Services Research, 20(1), 135. https://doi.org/10.1186/s12913-020-4968-2
31. Rushton, A. B., Verra, M. L., Emms, A., Heneghan, N. R., Falla, D., Reddington, M., Cole, A. A., Willems, P., Benneker, L., Selvey, D., Hutton, M., Heymans, M. W., & Staal, J. B. (2018). Development and validation of two clinical prediction models to inform clinical  decision-making for lumbar spinal fusion surgery for degenerative disorders and rehabilitation following surgery: protocol for a prospective observational study. BMJ Open, 8(5), e021078. https://doi.org/10.1136/bmjopen-2017-021078
32. Sharifi, B., McIntosh, G., Fisher, C., Jacobs, W. B., Johnson, M., Bailey, C. S., Christie, S., Charest-Morin, R., Paquet, J., Nataraj, A., Cadotte, D., Manson, N., Hall, H., Thomas, K. C., Rampersaud, Y. R., & Dea, N. (2019). Consultation and Surgical Wait Times in Cervical Spondylotic Myelopathy. The Canadian Journal of Neurological Sciences. Le Journal Canadien Des Sciences  Neurologiques, 46(4), 430–435. https://doi.org/10.1017/cjn.2019.34
33. Sørlie, A., Gulati, S., Giannadakis, C., Carlsen, S. M., Salvesen, Ø., Nygaard, Ø. P., & Solberg, T. K. (2016). Open discectomy vs microdiscectomy for lumbar disc herniation - a protocol for a  pragmatic comparative effectiveness study. F1000Research, 5, 2170. https://doi.org/10.12688/f1000research.9015.1
34. Spross, C., Aghayev, E., Kocher, R., Röder, C., Forster, T., & Kuelling, F. A. (2014). Incidence and risk factors for early adjacent vertebral fractures after balloon  kyphoplasty for osteoporotic fractures: analysis of the SWISSspine registry. European Spine Journal : Official Publication of the European Spine Society, the  European Spinal Deformity Society, and the European Section of the Cervical Spine Research Society, 23(6), 1332–1338. https://doi.org/10.1007/s00586-013-3052-6
35. Srinivas, S., Paquet, J., Bailey, C., Nataraj, A., Stratton, A., Johnson, M., Salo, P., Christie, S., Fisher, C., Hall, H., Manson, N., Rampersaud, Y. R., Thomas, K., McIntosh, G., & Dea, N. (2019). Effect of spinal decompression on back pain in lumbar spinal stenosis: a Canadian  Spine Outcomes Research Network (CSORN) study. The Spine Journal : Official Journal of the North American Spine Society, 19(6), 1001–1008. https://doi.org/10.1016/j.spinee.2019.01.003
36. Strömqvist, F., Jönsson, B., & Strömqvist, B. (2012). Dural lesions in decompression for lumbar spinal stenosis: incidence, risk factors  and effect on outcome. European Spine Journal : Official Publication of the European Spine Society, the  European Spinal Deformity Society, and the European Section of the Cervical Spine Research Society, 21(5), 825–828. https://doi.org/10.1007/s00586-011-2101-2
37. Strömqvist, F., Strömqvist, B., Jönsson, B., Gerdhem, P., & Karlsson, M. K. (2016). Lumbar disc herniation surgery in children: outcome and gender differences. European Spine Journal : Official Publication of the European Spine Society, the  European Spinal Deformity Society, and the European Section of the Cervical Spine Research Society, 25(2), 657–663. https://doi.org/10.1007/s00586-015-4149-x
38. Thomas, K., Faris, P., McIntosh, G., Manners, S., Abraham, E., Bailey, C. S., Paquet, J., Cadotte, D., Jacobs, W. B., Rampersaud, Y. R., Manson, N. A., Hall, H., & Fisher, C. G. (2019). Decompression alone vs. decompression plus fusion for claudication secondary to  lumbar spinal stenosis. The Spine Journal : Official Journal of the North American Spine Society, 19(10), 1633–1639. https://doi.org/10.1016/j.spinee.2019.06.003
39. Vinas-Rios, J. M., Medina-Govea, F. A., VON Beeg-Moreno, V., & Meyer, F. (2020). The degree of invasiveness has no influence on the rate of incidental durotomies in  surgery for lumbar spinal canal stenosis: data from the German spine registry. Journal of Neurosurgical Sciences, 64(6), 499–501. https://doi.org/10.23736/S0390-5616.18.04381-3
40. Vinas-Rios, J. M., Rauschmann, M., Medina-Govea, F., Sellei, R., Sobotke, R., & Arabmotlagh, M. (2020). There is no difference in perioperative results between posterior instrumentation  with and without interbody cage and debridement in primary spondylodiscitis in adults. A multicenter surveillance study from the German Spine Registry (DWG-Register). Journal of Neurosurgical Sciences. https://doi.org/10.23736/S0390-5616.19.04869-0
41. Vinas-Rios, J. M., Rauschmann, M., Sellei, R., Sanchez-Rodriguez, J. J., Meyer, F., & Arabmotlagh, M. (2019). Invasiveness has no influence on the rate of incidental durotomies in surgery for  multisegmental lumbar spinal canal stenosis (≥ 3 levels) with and without fusion. Analysis from the German Spine Registry data (DWG-Register). Journal of Neurosurgical Sciences. https://doi.org/10.23736/S0390-5616.19.04807-0
42. Vinas-Rios, J. M., Sanchez-Aguilar, M., Medina Govea, F. A., Von Beeg-Moreno, V., & Meyer, F. (2018). Incidence of early postoperative complications requiring surgical revision for  recurrent lumbar disc herniation after spinal surgery: a retrospective observational study of 9,310 patients from the German Spine Register. Patient Safety in Surgery, 12, 9. https://doi.org/10.1186/s13037-018-0157-1
43. Werner, D. A. T., Grotle, M., Gulati, S., Austevoll, I. M., Lønne, G., Nygaard, Ø. P., & Solberg, T. K. (2017). Criteria for failure and worsening after surgery for lumbar disc herniation: a  multicenter observational study based on data from the Norwegian Registry for Spine Surgery. European Spine Journal : Official Publication of the European Spine Society, the  European Spinal Deformity Society, and the European Section of the Cervical Spine Research Society, 26(10), 2650–2659. https://doi.org/10.1007/s00586-017-5185-5
44. Werner, D. A. T., Grotle, M., Gulati, S., Austevoll, I. M., Madsbu, M. A., Lønne, G., & Solberg, T. K. (2020). Can a Successful Outcome After Surgery for Lumbar Disc Herniation Be Defined by the  Oswestry Disability Index Raw Score? Global Spine Journal, 10(1), 47–54. https://doi.org/10.1177/2192568219851480
45. Yang, M. M. H., Riva-Cambrin, J., Cunningham, J., Jetté, N., Sajobi, T. T., Soroceanu, A., Lewkonia, P., Jacobs, W. B., & Casha, S. (2020). Development and validation of a clinical prediction score for poor postoperative  pain control following elective spine surgery. Journal of Neurosurgery. Spine, 1–10. https://doi.org/10.3171/2020.5.SPINE20347
46. Ziegler, D. S., Carreon, L., Andersen, M. O., & Jensen, R. K. (2019). The Association Between Preoperative MRI Findings and Surgical Revision Within Three  Years After Surgery for Lumbar Disc Herniation. Spine, 44(11), 818–825. <https://doi.org/10.1097/BRS.0000000000002947>
